# Bio‐Orthogonal Bacterial Reactor for Remission of Heavy Metal Poisoning and ROS Elimination

**DOI:** 10.1002/advs.201902500

**Published:** 2019-10-29

**Authors:** Pei Pan, Jin‐Xuan Fan, Xia‐Nan Wang, Jia‐Wei Wang, Di‐Wei Zheng, Han Cheng, Xian‐Zheng Zhang

**Affiliations:** ^1^ Key Laboratory of Biomedical Polymers of Ministry of Education and Department of Chemistry Wuhan University Wuhan 430072 P. R. China

**Keywords:** bacteria, bioreactor, Ceria, heavy metals, reactive oxygen species

## Abstract

Multitudinous industrial products in daily life put human health at risk of heavy metal exposure, and natural bacteria have displayed superior performance in bioadsorption and biodegradation of heavy metal. In this study, a bacteria‐based bioreactor is developed to precisely bioadsorb lead (Pb) ions, eliminate concomitant reactive oxygen species (ROS), and remit the injury of acute/chronic Pb poisoning. A nonpathogenic bacteria *Escherichia coli* MG1655 (Bac) is decorated with antioxidative cerium oxide nanoparticles (Ceria) on the surface through a bio‐orthogonal reaction, and the complex bioreactor could spontaneously aggregate in organs with high concentration of Pb. Furthermore, the excess Pb is bioadsorbed by bacteria and the concomitant ROS is eliminated by Ceria nanoparticles. In vitro and in vivo studies demonstrate that this integral biotic/abiotic hybrid bioreactor successfully realizes detoxication of Pb and reparation of injury, also accompanied with inappreciable side effects.

## Graphical abstract

A bio‐orthogonal bioreactor consisting of bacteria and Ceria is reported for heavy metal detoxication and subsequent reactive oxygen species (ROS) elimination. It is found that this bioreactor can achieve effective lead poisoning remission, and simultaneously eliminate excess ROS which continues to damage the body.

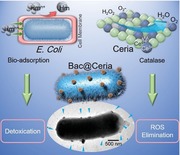

Exposure of heavy metals including cadmium (Cd), lead (Pb), and mercury (Hg) attracts significant concerns about human health.^1^, ^2^ Heavy metals could deactivate the proteins, create oxidative stress,^3^ disrupt endocrine system,^4^ and interfere with functions of essential cations^1^ to exert severe toxicities in multiple body systems.^1^, ^4^ As a representative, Pb is depicted as an essential inorganic toxin by the Centers for Disease Control and Prevention (CDC, USA), because it widely exists in automobile exhaust, industrial wastewater, and decorating materials,^1^, ^5^ which puts human health at risk of lead exposure especially for infants. Acute or chronic excessive intake of Pb causes severe injuries of poisoning to induce neurasthenia,^6^ cardiovascular,^7^ and kidney diseases,^1^ especially for intelligence development of kids.^8^, ^9^, ^10^ Nowadays, acute lead poisoning is generally treated with medication (2, 3‐dimercaptosuccinic acid, DMSA or edetate calcium disodium, EDTA)^11^ and hemodialysis^12^ to eliminate internal lead ion in clinical, though these treatments are accompanied with inevitable nonselection, strong side effects, and high expenses. Worse still, there is no efficient precaution for chronic lead poisoning in lead exposure districts.^11^, ^12^, ^13^ Consequently, it is quite necessary to develop a practical and economic therapy method for lead poisoning.

Noteworthy, as the oldest living creature worldwide, bacteria present remarkable resistance to heavy metals in nature.^14^, ^15^, ^16^ Profiting from diversified ingenious measures including adsorption,^17^ ion exchange,^18^ surface complexation,^16^ and precipitation,^19^, ^20^ bacteria could survive in harsh environment with excess heavy metals, and further bioadsorb metal ions to reduce concentration level. Based on these, bacteria are regarded as high‐efficiency bioreactors to sequester metal ions from ppm to ppb levels mainly by bioadsorption.^17^ Many nonpathogenic bacteria or probiotics have been widely modified by nanotechnology^21^ or edited by synthetic biology^22^, ^23^, ^24^, ^25^ for disease treatment. Therein, modification of macromolecules inside biological contexts site‐specifically and target‐selectively through bio‐orthogonal reaction was successful without disturbing active life activities in biological systems.^26^, ^27^ Bio‐orthogonal reaction was used to conjugate bacteria and inorganic nanoparticles through metabolic glycoengineering originated with unnatural sugars to construct a bioreactor for surface modification of bacteria. In comparison to conventional treatments, bacteria can accumulate to targeted organs with higher specificity,^28^ lower systemic toxicity, and greater efficiency,^29^ which means bacteria‐based bioreactors could be an intelligent antidote in lead‐enriched organs for lead poisoning.^30^ Additionally, lead poisoning not merely causes direct damages, but also breaks the balance between the production of reactive oxygen species (ROS) and the generation of antioxidants.^3^, ^13^ The imbalance results in excessive oxidative stress accompanied by persistent lipid membrane damage.^31^ Detoxification of reactive intermediates to reduce oxidative stress, further repair the resulting damage is as well as important.

Here, we reported an integral biotic/abiotic hybrid bioreactor to integrate lead detoxification and ROS elimination, and to further achieve remission of lead injury. Briefly, a nonpathogenic bacteria *Escherichia coli* MG1655 (*E. coli*, abbreviated to Bac), was bio‐orthogonal modified with antioxidative cerium oxide nanoparticles (Ceria) on the surface. Ceria is a universal antioxidative reagent which possesses commendable antioxidant activity at physiological pH,^32^, ^33^ and the bacteria could actively carry Ceria to accumulate in a variety of organs with high concentration of lead. The excess toxic metals were mainly bioadsorbed according to metal resistance of bacteria, and the imbalance of oxidative stress was simultaneously repaired by Ceria. In vitro and in vivo consequences demonstrated that the hybrid bioreactor was safe enough for biomedical application, and exhibited remarkable lesion targeting, lead elimination, and antioxidative efficacy.

Substantial studies have demonstrated that lead exposure would induce severe harm to human health, especially to children.^1^, ^9^ To investigate the distribution of lead exposure in different districts, the statistical data of blood lead level (BLL) from several children in different USA states were detected and are shown in Figure S1 in the Supporting Information, which sources from CDC. Also, the distribution of lead‐poisoning patients in the USA was collected and shown, which revealed lead exposure would cause potential hazard to human health seriously (Figure S2, Supporting Information). Up to date, there is no secure and valid detoxicant for lead degradation yet. However, anecdotal evidence suggests that microorganism was capable to obliterate toxic heavy metals in wastewater,^34^ but the efficiency to clear them away is various in different bacteria. Based on this, utilizing bacteria to degrade heavy metals could be considered. It is significant to screen a nonpathogenic bacterium for obliterating toxic heavy metals alternatively. The lead detoxified and ROS eliminated bioreactor based on bacteria, the focal therapy procedure, and its degradation mechanism of heavy metals is illustrated in **Figure** **1**a. In order to screen the most valid natural bacteria for heavy metals obliterating, *Bacillus subtilis* (*B. subtilis*), *E. coli*, *Lactobacillus acidophilus* (*L. acidophilus*), *Clostridium butyricum* (*C. butyricum*), and *Bacillus thuringiensis* (*B. thuringiensis*) were used to degrade Cu^2+^, Pb^2+^, and Cd^2+^ in Figure 1b. It was observed that the efficiency of *E. coli* to eliminate lead and cadmium was remarkable by bioadsorption and biomineralization possibly, which indicated *E. coli* could remove lead selectively. As a representative, lead was selected to evaluate the degradation efficiency of bioreactor, and the degradation rate from various concentrations of lead detoxified by *E. coli* was measured in Figure S3 in the Supporting Information. *E. coli* possessed great properties of lead‐resistance and lead‐detoxification from a range of concentrations of lead. Thus, we chose *E. coli* strain MG1655, a nonpathogenic lipopolysaccharide (LPS)‐free bacteria strain to degrade heavy metals, such as lead, cadmium, etc.

**Figure 1:**
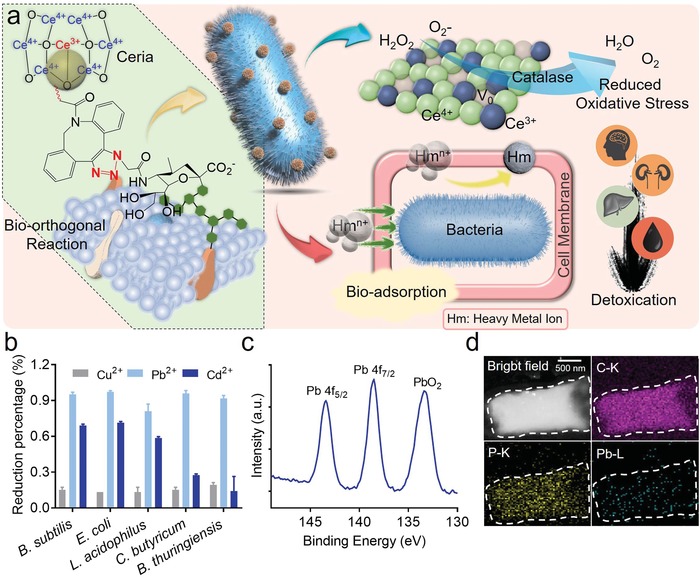
a) The scheme of bacteria‐based bioreactor and its mechanism for the detoxification of heavy metals and ROS. b) Effect of biodegradation rate of different heavy metals by various bacteria. c) XPS spectrum of Pb (4f_5/2_), Pb (4f_7/2_), and PbO_2_ at the surface of *E. coli* after bacteria treatment. d) EDX mapping images of the distribution of elemental carbon, phosphorus, and lead at the surface of bacteria after co‐culturing with 50 × 10^−6^ m lead.

In addition, mounting evidence suggests that the mechanism of heavy metal degradation by bacteria was due to bioadsorption^17^ and biomineralization.^19^ During lead exposure, the process of Pb^2+^ converted into Pb and PbO_2_ after being treated with Bac was measured with X‐ray photoelectron spectroscopy (XPS) analysis (Figure 1c), which revealed that lead was partly cleared away through biomineralization. Next, after being co‐cultivated with different concentrations of lead (50 × 10^−6^ and 500 × 10^−6^ m), zeta potentials of Bac were measured at different time points (Figure S4, Supporting Information). The values were increased as time prolonged, which revealed Pb^2+^ was degraded by biomineralization. For further proof, we observed the distribution of Pb on the surface of Bac with energy‐dispersive X‐ray spectroscopy (EDX) mapping analysis, and lead element was shown on the surface of Bac which illustrated the removal of lead was through bioadsorption and biomineralization by Bac treatment (Figure 1d). Besides, bacterial debris of Bac was prepared by heating temperature. The lead solution was treated by the bacterial debris of Bac or Bac, after which the lead reduction rates were measured by inductively coupled plasma‐mass spectrometry (ICP‐MS). As outlined in Figure S5 in the Supporting Information, there is no significant change of lead reduction rates which indicated that Pb^2+^ was degraded by bioadsorption either. Additionally, the viability of Bac was slightly influenced after lead exposure, suggesting Bac had strong tolerance to lead (Figure S6, Supporting Information). Also, the sodium dodecyl sulfate‐polyacrylamide gel electrophoresis (SDS‐PAGE) showed the mild transformation of the proteins in Bac (Figure S7, Supporting Information). Collectively, these results manifested that Bac with heavy metal tolerance exerted predominant capability for heavy metals elimination through bioadsorption and biomineralization.

However, Bac cannot have the ability to reduce oxidative stress levels. Nonetheless, lead exposure would induce elevated levels of oxidative stress,^3^ accounting for it is critical to find a component with excellent antioxidant capacity. Plentiful studies have confirmed that the antioxidative property and biocompatibility of Ceria were potent.^32^, ^33^, ^35^ Hence, Ceria nanoparticles were synthesized for oxidative stress relief to achieve the restoration of lead injury. In terms of transmission electron microscope (TEM) images (**Figure** **2**a) and dynamic light scattering (DLS) analysis (Figure 2b), the uniform size and structure of Ceria were revealed and the hydrate size of Ceria was about 50 nm. In order to construct the bacteria‐based bioreactor, Bac was labeled by 2‐azido‐2‐deoxy‐d‐glucose for preparing N_3_‐Bac, and Ceria nanoparticles were modified by azo‐dibenzocyclooctyne (DBCO) for obtaining DBCO‐Ceria. Through bio‐orthogonal click reaction, Ceria was anchored on the surface of Bac covalently and a bacteria‐based biotic–abiotic hybrid bioreactor (Bac@Ceria) was constructed (Figure S8, Supporting Information). The bioreactor was equipped with predominant ability to accelerate the heavy metal and ROS biodegradation process for maintaining the physiological balance of body.

**Figure 2:**
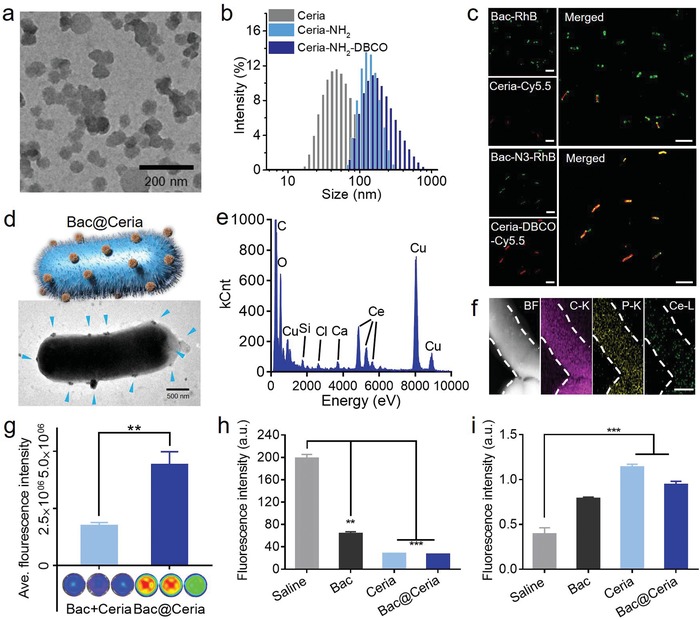
a) TEM image of Ceria (Scale bar: 200 nm). b) Hydrodynamic sizes of Ceria, Ceria‐NH_2_, and Ceria‐NH_2_‐DBCO. c) In vitro Ceria‐DBCO binding assay to bacteria‐N_3_/bacteria. Bacteria were marked by RhB, and Ceria nanoparticles were labeled by Cy5.5. (Scale bar: 2 µm.) d) TEM image of Bac@Ceria. (Scale bar: 500 nm.) e) Distribution of elements on the surface of Bac@Ceria detected by EDX. f) EDX elemental mapping of Bac@Ceria, such as carbon, phosphorus, and cerium. (Scale bar: 500 nm.) g) Agarose hydrogel embed‐bacteria with/without azide modified for depicting the ability of bio‐orthogonal between Ceria‐DBCO and bacteria‐N_3_. h) The elimination efficiency of •$O_{2}^{-}$ detected by TA. i) The elimination efficiency of H_2_O_2_ detected by MB. Significance between every two groups was calculated using unpaired two‐tailed Student's *t*‐test unless otherwise indicated. *P < 0.05, **P < 0.01, ***P < 0.001. The values are presented as mean ± SD.

In order to confirm the extent of bio‐orthogonal chemistry, Bac and N_3_‐Bac were labeled by rhodamine B (RhB), and Ceria and DBCO‐Ceria were labeled by Cy5.5 at meanwhile. Bac and N_3_‐Bac were added into Ceria and DBCO‐Ceria, respectively. Cy5.5‐labeled DBCO‐Ceria was obviously observed to stick on the surface of RhB‐labeled N_3_‐Bac, and weak Cy5.5 fluorescence was found on the surface of Bac (Figure 2c), which identified the successful construction of Bac@Ceria from DBCO‐Ceria and N_3_‐Bac by bio‐orthogonal chemistry. The same consequence was verified in TEM, TEM/EDX, and EDX mapping analysis (Figure 2d–f). As shown in Figure 2g, the fluorescence of DBCO‐Ceria in N_3_‐Bac immobilized hydrogels was much stronger, which indicated that the bio‐orthogonal reaction between DBCO‐Ceria and N_3_‐Bac was under stable condition. Additionally, Fourier transform infrared (FT‐IR) spectrum of Ceria, Ceria‐NH_2_, DBCO‐Ceria, Bac, and Bac@Ceria (Figure S9, Supporting Information) and the change of zeta potentials (Figure S10, Supporting Information) in Ceria, Ceria‐NH_2_, Ceria‐DBCO, Bac, and Bac@Ceria also showed the synthesis of components in the bioreactor and the facilitative effect of bio‐orthogonal click reaction. Based‐on these results, the construction of Bac@Ceira biotic/abiotic hybrid was successful. Then, the modification would not decrease cell ability of bacteria that was identified. Bac and Bac@Ceria were spread on LB agar plates, and the quantity of colonies demonstrated that the modification of Ceria would not impact the cell viability (Figure S11a, Supporting Information). The OD600 changes of the Bac and Bac@Ceria also certified the safety of Ceria to Bac (Figure S11b, Supporting Information).

We then investigated the antioxidative competence of Bac@Ceria. As a constituent of the bioreactor, the function of Ceria nanoparticles was responsible for an antioxidant to eliminate ROS. Among them, the composition of Ceria was not only cerium (III) oxide but also cerium (IV) oxide. Cerium (III) was developed as superoxide dismutase (SOD)‐mimetic to remove •$O_{2}^{-}$, whereas cerium (IV) was used as catalase (CAT)‐mimetic for H_2_O_2_ oxidizing.^33^ The valence states of Ceria on the surface of Bac@Ceria were indicated by XPS analysis (Figure S12, Supporting Information). The valence states of cerium (III) peaks were at 889.1 and 907.3 eV, and cerium (IV) peaks were at 882.1, 898, 900.75, and 916.65 eV, and the ratio of Ce^4+^/Ce^3+^ was 3.033. Additionally, the ROS catalytic efficiency of Bac@Ceria was determined by terephthalic acid (TA) and methylene blue (MB), individually. With the presence of H_2_O_2_, TA could be oxidized to 2‐hydroxyterephthalic acid which possessed a strong fluorescent peak at 425 nm.^36^ Compared with negative control, Bac, Ceria, and Bac@Ceria showed strong eliminated capacity after being incubated with H_2_O_2_ (Figure 2h). For further exploration, the consequences showed that a series of concentrations of Bac@Ceria could degrade H_2_O_2_ after co‐incubation between various concentrations of Bac@Ceria and H_2_O_2_ (Figure S13, Supporting Information). Similarly, degradation of MB also indicated the same antioxidative ability of Bac@Ceria (Figure 2i). These phenomena depicted that the biotic/abiotic hybrid system exhibited superior capacity of the elimination of ROS to compete oxidative stress after lead exposure.

Then, the biological effects of components of Bac@Ceria were studied in COS7 cells (African green monkey kidney cells) and 3T3 cells (3T3 mouse fibroblasts). To evaluate the cell viability in vitro, both Bac, Ceria and Bac@Ceria were incubated with COS7 cells and 3T3 cells for 24 h, respectively. After being treated by various concentrations of Ceria in COS7 and 3T3 cells for 24 h, the cell viabilities still remained (>95%) (Figure S14, Supporting Information), which indicated the excellent security of Ceria. Furthermore, the cell viabilities were assessed by MTT after being co‐cultivated with Bac and Bac@Ceria for 24 h in utilizing transwell chamber. **Figure** **3**a,b also illustrates the less cytotoxic effects of Bac and Bac@Ceria, individually. Next, the ROS (caused by H_2_O_2_) eliminated ability of the biotic/abiotic hybrid system was investigated in vitro (Figure 3c). Noticeably, when exposed under H_2_O_2_, almost all of the cells were died. Meanwhile, Ceria and Bac@Ceria could remarkably degrade H_2_O_2_ and protect cells from oxidative damage. Also, the lead detoxification capabilities were assessed in COS7 and 3T3 cells. After 24 h lead exposure, bacteria showed obviously lead‐resistant and could detoxify lead markedly to protect cells from lead destruction (Figure 3d).

**Figure 3:**
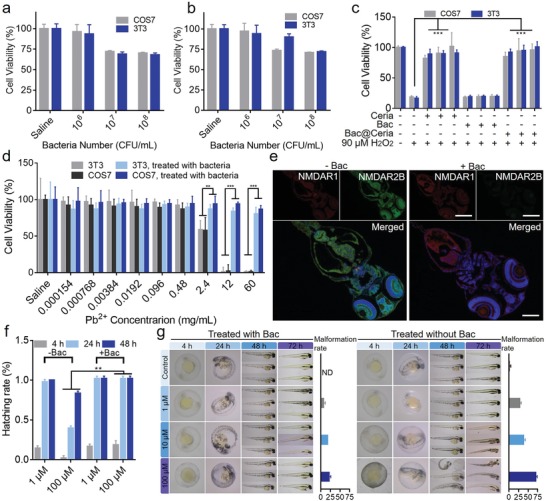
a) In vitro viability of COS7 and 3T3 cells after incubation with various concentration of bacteria for 24 h. b) In vitro viability of COS7 and 3T3 cells after incubation with various concentrations of Bac@Ceria for 24 h. c) In vitro antioxidative capacity of Ceria/Bac/Bac@Ceria in COS7 and 3T3 cells. d) In vitro lead biodegradation capacity with/without Bac treatment in COS7 and 3T3 cells. e) The mRNA expression of NMDA receptors in zebrafish larvae after lead exposure with/without bacteria treatment. (Scale bar: 100 µm.) f) Hatching rate of zebrafish embryo after various concentrations of lead with/without bacteria treatment. g) Images and malformation rate of zebrafish larvae after lead exposure with/without bacteria treatment. Significance between every two groups was calculated using unpaired two‐tailed Student's *t*‐test unless otherwise indicated. *P < 0.05, **P < 0.01, ***P < 0.001. The values are presented as mean ± SD.

Furthermore, some researches showed that lead exposure would cause the infants' nervous system damage and physical deformity.^8^ Previous studies revealed *N*‐methyl‐*D*‐aspartiate (NMDA) receptors were related with nerve and could medicate learning ability, memory, neuronal development, and excitatory synaptic neurotransmission by regulating their subcellular structure and function.^37^ NMDA receptors were assembly composited by three kinds of isoforms, such as NR1 subunits, NR2 subunits, and NR3 subunits.^38^ Ordinarily, NR1 and NR2B would overexpress at the early postnatal time.^37^, ^38^ To study the behavior and neuron protective effects of this bioreactor system under lead exposure, we used zebrafish embryos as the model animal. The zebrafish embryos were incubated under the basic culture medium with lead solution after being bacteria treated or not. Thus, the mRNA expression of NMDA receptors was measured by immunofluorescence staining after zebrafish larvae hatching from embryos. Figure 3e illustrates that the NMDAR2 expression of zebrafish with the lead exposure after being treated by bacteria was less expressed than the lead exposure group without bacteria treatment, supposing that lead exposure might delay the possess of NMDAR1 exchanged to NMDAR2. Afterward, the living status of zebrafish embryos and larvae were surveilled at different time points. As illustrated in Figure 3f,g, lead exposure would decrease the hatching rate and increase the malformation rate of zebrafish embryos, but bacteria treatment could protect the embryos from dying and malformation. These results strongly showed the remarkable capability of bacteria to degrade lead.

Subsequently, the in vivo lead detoxification and ROS elimination effects were evaluated in chronic poisoning‐mice and acute poisoning‐mice models. As shown in **Figure** **4**a, Bac@Ceria was orally administrated into chronic poisoning‐mice. After being gavaged Bac per day or not, the feces were collected and the proportion of top ten abundance bacterial genera were measured by 16S ribosomal DNA identification to identify the colonization of Bac. It is visible that though the contents of intestinal microflora were changed at the first few days, it restituted in a week (Figure 4b). In clinic, DMSA and EDTA are two major drugs for clinical treatment, mainly through chelation, after heavy metal poisoning.^11^ However, there were some side effects, for example, EDTA would injure nervous system seriously after several days treatment, and DMSA would cause oxidative stress to damage normal tissues.^11^ Thus, an effective method to eliminate heavy metals, repair injury‐lesion, and protect normal tissues from being damaged was needed. Kunming mice were used in this study. Newborn Kunming mice were randomly divided into four groups (*n* = 12), all of them were obtained lead exposure by breast‐feeding, while mouse mothers were gavaged lead solution (1 mg kg^−1^) at the first 7 days. After the first 7 days, the newborn mice were gavaged lead solution (1 mg kg^−1^) individually. For treatment, three of these groups were orally administered with EDTA (i.v., 15 mg kg^−1^), DMSA (oral, 15 mg kg^−1^), and Bac@Ceria (oral, 50 µg kg^−1^, 10^10^ CFU), respectively. During the treatment, Bac@Ceria was depicted remarkable efficiency for the precaution, remission, and treatment of heavy metal poisoning. To evaluate the change of oxidative stress after treatment in vivo, myeloperoxidase (MPO) in mice intestine tissues were measured. MPO was a kind of leukocyte‐derived heme protein to generate a series of ROS,^39^ which was related with the ROS level and body injury. Figure 4c reveals that Bac@Ceria could significantly decrease the content of MPO to reduce ROS level in chronic poisoning‐mice intestine. Additionally, the lead concentrations of blood and the main organs were measured by ICP‐MS to monitor the lead degradation effect of Bac@Ceria. As shown in Figure 4d,e, Bac@Ceria possessed distinguished lead detoxification capacity in vivo. Besides, the survival rates indicated that Bac@Ceria could delay the life of lead exposed mice (Figure 4f), and the body weights showed the negligible side effects of this treatment (Figure S15, Supporting Information). To evaluate the brain and neuro protection ability of Bac@Ceria under lead exposure, several chronic poisoning‐mice were chosen for Morris water maze (MWM) analysis to assess the learning and memory of lead exposed mice. MWM performance was a key way to investigate the hippocampal circuitry,^40^ because long‐term potentiation and NMDA receptor function were related to its performance.^37^ Figure 4g depicts that Bac@Ceria could make the learning and memory of mice maintained compared with saline group. Furthermore, negligible toxicity and brain injury of Bac@Ceria were observed by hematoxylin‐eosin (H&E) staining, and the developmental and health status of Bac@Ceria were demonstrated more distinguishably (Figure 4h; Figure S16, Supporting Information).

**Figure 4:**
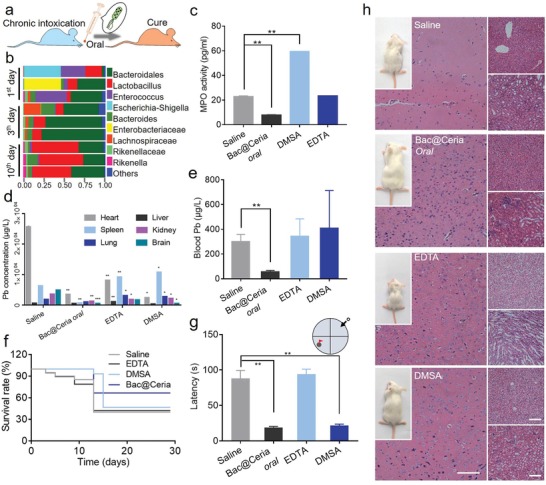
a) Timeline of treatments of Bac@Ceria in chronic poisoning‐mice. b) Distribution of the top ten bacteria community types in murine feces after *E. coli* treatment at different time points. c) The content of myeloperoxidase (MPO) in chronic poisoning‐mice intestine tissues after being treated with Bac@Ceria, DMSA, and EDTA. d) The content of lead in main organs of chronic poisoning‐mice after being treated with Bac@Ceria, DMSA, and EDTA. e) The content of lead in blood of chronic poisoning‐mice after being treated with Bac@Ceria, DMSA, and EDTA. f) Survival curves of lead exposure chronic poisoning‐mice after different treatments. g) The latency to reach the goal in chronic poisoning‐mice after being treated with saline, Bac@Ceria, DMSA, and EDTA. h) H&E staining of brain, liver, and spleen tissues in chronic poisoning‐mice after different treatments (Scale bar: 100 µm). Significance between every two groups was calculated using unpaired two‐tailed Student's *t*‐test unless otherwise indicated. *P < 0.05, **P < 0.01, ***P < 0.001. The values are presented as mean ± SD.

In addition, previous studies revealed that lead would accumulate in kidney or liver causing critical damage after lead exposure.^1^ However, Ceria lack specificity to accurately aggregate into kidneys, but some bacteria possessed this capability. Thus, the in vivo biodistribution of Bac@Ceria was evaluated. Mice were randomly divided into four groups (*n* = 3). Cy5.5‐labeled Ceria, 1,1‐dioctadecyl‐3,3,3,3‐tetramethylindotricarbocyanine iodide (DiR iodide)‐labeled Bac, and DiR‐labeled Bac@Ceria were orally administered into mice, meanwhile Bac@Ceria was also injected into mice at another group. As shown in **Figure** **5**a, Bac@Ceria was found to target liver and spleen with time prolonging. To the contrary, negligible liver or kidney retention of Ceria was found.

**Figure 5:**
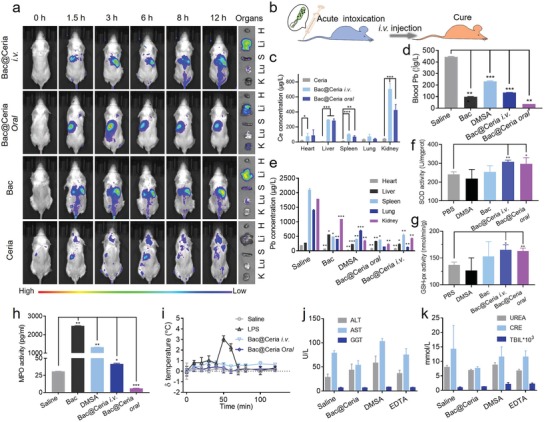
a) In vivo and ex vivo optical images of mice after the injection of Bac, Ceria, and Bac@Ceria, and oral administration of Bac@Ceria. b) Timeline of treatments of Bac@Ceria in acute poisoning‐mice. c) ICP‐MS detection of Ce content in different organs. d) The content of lead in blood in acute poisoning‐mice after being treated with Bac, DMSA, Bac@Ceria i.v., and Bac@Ceria oral. e) The content of lead in main organs of acute poisoning‐mice after being treated with Bac, DMSA, Bac@Ceria i.v., and Bac@Ceria oral. f–h) The contents of SOD, GSH‐px, and MPO in acute poisoning‐mice intestine tissues after being treated with Bac, DMSA, Bac@Ceria i.v., and Bac@Ceria oral. i) The change of body temperature curve of mice after treatment of Bac@Ceria. j,k) Blood biochemistry examination indexes of lead exposure mice after different treatments. Significance between every two groups was calculated using unpaired two‐tailed Student's *t*‐test unless otherwise indicated. *P < 0.05, **P < 0.01, ***P < 0.001. The values are presented as mean ± SD.

Collectively, the lead detoxification and damage reparation effects were also certified in acute poisoning‐mice models (Figure 5b), and the ICP‐MS detection of Ce content in different organs is shown in Figure 5c. Acute poisoning‐mice were divided into five groups randomly (*n* = 6). All of these groups were used to establish acute poisoning‐mice model by orally administered lead (100 mg kg^−1^). Mice of three groups were orally administered with normal saline, Bac (oral, 10^10^ CFU), and Bac@Ceria (oral, 50 µg kg^−1^, 10^10^ CFU). And another two groups were injected with EDTA (i.v., 30 mg kg^−1^) and Bac@Ceria (i.v., 50 µg kg^−1^, 10^7^ CFU). The lead concentrations of blood and main organs were measured by ICP‐MS, and Figure 5d,e illustrates that orally administrated Bac@Ceria could degrade lead obviously. After various treatments, mice were sacrificed, and the SOD (Figure 5f), glutathione peroxidase (GSH‐px, Figure 5g), and MPO (Figure 5h) values were measured, which indicated that this treatment could be anti‐inflammatory. The same as MPO, SOD and GSH‐px were also two of the significant markers for ROS production in vivo .^41^ Then, negligible side effects of Bac@Ceria were displayed by body weight (Figure S17, Supporting Information). This hybrid bioreactor also could repair the body injury in acute poisoning‐mice at intestine by H&E staining (Figure S18, Supporting Information).

Furthermore, the biosafety of Bac@Ceria was measured. We verified mice body temperature after the treatment, and the change of body temperature was normal, which indicated that Bac@Ceria at a dose of 10^10^ per mouse would not cause sepsis (Figure 5i). Besides, blood biochemistry was tested to analyze the side effects of this therapy. These results showed that urea, creatinine (CRE), alanine aminotransferase (ALT), aspartate aminotransferase (AST), glutamyl transpeptadase (GGT), and total bilirubin (TBIL) were in normal range (Figure 5j,k). In addition, the C‐reactive protein (CRP) and procalcitonin (PCT) were vital markers for systemic infection .^42^ The concentrations of serum CRP and PCT were analyzed after saline, Bac (oral, 10^10^ CFU), Bac@Ceria (i.v., 50 µg kg^−1^, 10^10^ CFU), and Bac@Ceria (oral, 50 µg kg^−1^, 10^10^ CFU) administration, which identified the merely secondary action and the splendid biosecurity of Bac and Bac@Ceria (Figure S19, Supporting Information).

In conclusion, a hybrid bioreactor Bac@Ceria for heavy metal degradation and ROS elimination was constructed. In Bac@Ceria, nonpathogenic bacteria were applied as smart robot for targeting and detoxifying toward nidus. Ceria acted as an antioxidant for ROS elimination and body repair. It was found that Bac@Ceria achieved excellent heavy metal degradation and anti‐inflammatory effects with both intravenous injection and oral administration. Compared with classical treatments of heavy metal poisoning including hematodialysis and drug therapy, this self‐powered bioreactor, with targeting and penetrating ability, significantly showed higher efficiency and lower side effects.

## Experimental Section

Methods are available in the Supporting Information.

## Conflict of Interest

The authors declare no conflict of interest.

## Supporting information

Supporting Information
