# Supplementary material for: Bio‐Orthogonal Bacterial Reactor for Remission of Heavy Metal Poisoning and ROS Elimination
Source: Adv Sci (Weinh). 2019 Oct 29;6(24):1902500. doi: 10.1002/advs.201902500 (PMC6918106; doi:10.1002/advs.201902500)
Supplement: Supplementary file 1 — Supporting Information [file ADVS-6-1902500-s001.pdf]

Copyright WILEY-VCH Verlag GmbH & Co. KGaA, 69469 Weinheim, Germany, 2019.

ADVANCED SCIENCE
Open Access

Supporting Information

for *Adv. Sci.*, DOI: 10.1002/advs.201902500

Bio-Orthogonal Bacterial Reactor for Remission of Heavy Metal Poisoning and ROS Elimination

*Pei Pan, Jin-Xuan Fan, Xia-Nan Wang, Jia-Wei Wang, Di-Wei Zheng, Han Cheng, and Xian-Zheng Zhang**

Supporting Information

of

Bio-Orthogonal Bacterial Reactor for Remission of Heavy Metal Poisoning and ROS Elimination

Pei Pan[§, †], Jin-Xuan Fan[§, †], Xia-Nan Wang[†], Jia-Wei Wang[†], Di-Wei Zheng[†], Han Cheng[†] and Xian-Zheng Zhang[*, †]

P. Pan, J. X. Fan, X. N. Wang, J. W. Wang, D. W. Zheng, Dr. H. Cheng, Prof. Dr. X. Z. Zhang

[†] Key Laboratory of Biomedical Polymers of Ministry of Education

& Department of Chemistry

Wuhan University

Wuhan 430072, P. R. China

* E-mail: xz-zhang@whu.edu.cn

[§]P. Pan and J. X. Fan contributed equally to this work.

Experiment section

Materials. 1,1-dioctadecyl-3,3,3,3- tetramethylindotricarbocyanine iodide (DiR iodide) and 3-[4,5-Dimethylthiazol-2-yl]-2,5-diphenyltetrazolium-bromide (MTT) were supplied by Beyotime Biotechnology Co. Ltd. (China). Agar and Luria-Bertani (LB) broth were purchased from HuanKai Microbial Co. Ltd. (China). Brain Heart Infusion (BHI) broth was obtained from Qingdao Hope Biotechnology (China). DBCO-PEG_{2000}-NHS, cadmium nitrate tetrahydrate, lead nitrate, copper nitrate, terephthalic acid (TA) and 3-Aminopropyltriethoxysilane were provided by Aladdin (Shanghai, China). 2-Azido-2-deoxy-D-glucose was purchased from Toronto Research Chemicals Rd., Toronto, Ontario Canada. Edetate calcium disodium (EDTA) and 2, 3-dimercaptosuccinic acid (DMSA) was supplied by Sigma-Aldrich Co. LLC. Methylene blue (MB) was purchased by TCI (Shanghai) Development Company (Shanghai, China). The PCT concentrations in serum were measured by a mouse PCT ELISA kit (Eaivelly, Shanghai, China). The serum CRP concentrations were analyzed by a mouse CRP ELISA kit (Bio-Swamp life science, China).

Cell lines. COS7 cells (African green monkey kidney cells), 3T3 cells (3T3 mouse fibroblasts) were purchased from China Center for Type Culture Collection (CCTCC).

Bacterial strains. *Escherichia coli* (*E. coli*, strain MG1655), *Bacillus subtilis* (ATCC 6051), *Bacillus thuringiensis* (ATCC 10792), and *Clostridium butyricum* (ATCC 19398) strains were collected from the American Type Culture Collection (ATCC). *Lactobacillus acidophilus* (CCTCC AB 2010208) were obtained from China Center for Type Culture Collection (CCTCC). LB broth and BHI broth were used for the bacteria culture.

Bacteria screening. The heavy metal removing ability of several bacterial strains, such as *Escherichia coli* (*E. coli*), *Bacillus subtilis* (*B. subtilis*), *Bacillus thuringiensis* (*B. thuringiensis*), *Clostridium butyricum* (*C. butyricum*), and *Lactobacillus acidophilus* (*L. acidophilus*) was analyzed. All strains were incubated for 18 h, and then *E. coli* (10^8 CFU mL^{-1}), *B. subtilis* (10^8 CFU mL^{-1}), *B. thuringiensis* (10^8 CFU mL^{-1}), *C. butyricum* (10^8 CFU mL^{-1})

and *L. acidophilus* (10^8 CFU mL^{-1}) were centrifuged at 3920 g for 3 min individually. Respectively, the cell pellets were divided into 3 parts. One of them was resuspended in normal saline containing 40 μmol L^{-1} Cu^{2+} as copper sulfate. Another of them was resuspended in normal saline containing 40 μmol L^{-1} Pb^{2+} as lead nitrate. The third of them was resuspended in normal saline containing 40 μmol L^{-1} Cd^{2+} as cadmium nitrate. The heavy metal reduction ability was assessed by inductively coupled plasma atomic emission spectroscopy (ICP-AES, IRIS Intrepid II XSP).

Synthesis of Ceria. 1 mmol L^{-1} cerium (III) acetate and 12 mmol L^{-1} oleylamine were mixed into 15 mL of xylene. The solution was heated to 90 °C and added DI water (1 mL) rapidly after stirred violently for 12 h at room temperature. The mixture was heated to 90 °C for 3 hours, and then cooled into 25 °C. The Ceria nanoparticles were decentralized in acetone and separated by centrifugation. The sediment was dispersed in chloroform. Ceria nanoparticles were storage at a vacuum oven (80 °C) to remove the chloroform.

Synthesis of DBCO-Ceria. 200 mg L^{-1} ceria nanoparticles were mixed with 2 g L^{-1} 3-aminopropyltriethoxysilane for 12 h at room temperature. The mixture was centrifuged at 7840 g for 20 min and washed by DI water for three times, and the precipitation was resuspended in ultrapure water to obtain Ceria-NH_2. 20 mg DBCO-PEG_{2000}-NHS was dissolved in ultrapure water and added into the above reaction mixture and stirred at for 24 h room temperature.

Synthesis of N_3-Bac. 1 mg 2-azido-2-deoxy-D-glucose was added into 10 mL LB broth and co-cultivated with *E. coli* (10^8 CFU mL^{-1}) overnight.

Synthesis of Bac@Ceria. DBCO-Ceria (50 μg mL^{-1}) was added into the solution which contained N_3-Bac (10^8 CFU mL^{-1}) for 6 h at 37 °C. The mixture was centrifuged at 6000 rpm for 3 min and the sediment was washed by DI water for 3 times.

In vitro cytotoxicity assay of Ceria to Bac. Bac was cultivated in LB broth at 37 °C. DBCO-Ceria (50 μg mL^{-1}) and N_3-Bac (10^8 CFU mL^{-1}) were co-cultivated in LB broth at 37 °C for 6

h to obtain Bac@Ceria. Once the OD600 of culture reached 0.8, Bac and Bac@Ceria were diluted for 100 folds. Then, Bac and Bac@Ceria were cultured in LB at 37 ^{o}C. The OD600 of them were measured at different time points by a microplate reader (Bio-Rad, Model 550, USA).

Hydroxyl radical scavenging assay of Bac@Ceria. 1.8 mmol L^{-1} $FeSO_4$ and 5 mmol L^{-1} H_2O_2 were mixed for •OH generation. 0.005 mg mL^{-1} methylene blue (MB) was added into the above mixture and co- cultivated with Bac (10^8 CFU mL^{-1}), Ceria (50 μg mL^{-1}), or Bac@Ceria (50 μg mL^{-1}) for 5 h at 37 □, individually. Then the absorbance of the mixtures was measured at 664 nm by Ultraviolet & visible diffuse reflectance spectroscopy (Lambda Bio40).

Hydrogen peroxide scavenging assay of Bac@Ceria. H_2O_2 (9 μmol L^{-1}) was mixed with Bac (10^8 CFU mL^{-1}), Ceria (50 μg mL^{-1}), or Bac@Ceria (50 μg mL^{-1}) for 6 h at 37 □, individually. Terephthalic acid (TA) was added for ROS detection. Then the fluorescence was measured at 425 nm by UV-vis spectroscopy.

Self-assembly of bacteria-nanoparticles conjugates assay. Ceria and Ceria-DBCO were modified by Cy5.5-NHS. Bac or Bac-N_3 were labeled with RhB and mixed with Ceria or Ceria-DBCO for 4 h. The mixture was washed by DI water for three times. Subsequently, the fluorescence was observed by a super-resolution microscope (LEICA (DMi8)).

In vitro cytotoxicity assay. COS7 cells and 3T3 cells were seeded into 96-well plates with a density of 1 × 10^4 cells per well. 100 μL of medium were added to each well, which was supplemented with 10% fetal bovine serum (FBS). Cells were cultured under the atmosphere of 5% CO_2 for 24 h at 37 □. The medium was refreshed and followed by adding Ceria nanoparticles with different concentrations (0-500 μg mL^{-1}). After co-incubating for 24 h, the medium was added with 10 μL 3-(4,5-dimethyl-2-thiazolyl)-2,5-diphenyl-2-Htetrazolium bromide (MTT, 5 mg mL^{-1}) and co-incubated for another 4 h, then the medium was discarded and 150 μL of DMSO was added. The absorbance at 570 nm was measured by a microplate

reader.

COS7 cells and 3T3 cells were seeded into 24-well plates with a density of 1 × 10^5 cells per well. Bacteria (10^8 CFU mL^{-1}) and Bac@Ceria (50 μg mL^{-1}, 10^8 CFU mL^{-1}) were co-cultivated with these cell lines for 24 h by using Transwell chamber (Corning Costar, USA). Then, the cell viability was measured by MTT assay.

In vitro lead degradation effects of Bac@Ceria. COS7 cells and 3T3 cells were seeded into 96-well plates with a density of 1 × 10^4 cells per well. Different concentrations of Pb^{2+} solution (0-60 mg mL^{-1}) were treated with or without Bac@Ceria (50 μg mL^{-1}). After that, these mixtures were centrifuged at 3920 g for 3 min, the supernatants were added to the cell medium and co-incubated with cells for 24h. Then, the cell viability was measured by MTT assay.

In vitro anti-oxidative effects of Bac@Ceria. COS7 cells and 3T3 cells were seeded into 96-well plates with a density of 1 × 10^4 cells per well. Various concentrations of Bac (10^7-10^9 CFU mL^{-1}), Bac@Ceria (10^7-10^9 CFU mL^{-1}) and Ceria (50 μg mL^{-1}) were added into 90 μmol L^{-1} H_2O_2 and incubated for 6 h. These mixtures were centrifuged at 3920 g for 3 min. These cell lines were exposed to various concentrations of the supernatants for 24 h. Then, the cell viability was measured by MTT assay.

The lead degradation effects of Bac on zebrafish embryos and larvae. The aquaculture water for zebrafish culture containing 0.5 mM KCl, 15 mM NaCl, 1.0 mM $MgSO_4$, 0.05 mM Na_2HPO_4, 0.15 mM KH_2PO_4 and 0.7 mM $NaHCO_3$ was used for zebrafish embryos incubation. Wild-type (AB strain) zebrafish embryos obtained from Institute of Hydrobiology (Chinese Academy of Sciences), were cultured in aquaculture water containing various concentrations of Pb^{2+} (0-100 μM) after being treated with/without bacteria in a light-dark cycle at approximately 28 ^{o}C. At 0 h, 4 h, 24 h, 48 h, 72 h post-fertilization, hatched embryos were collected and observed under stereoscopic microscope. The hatch rate and malformation rate were also calculated and compared. At the end of incubation, the zebrafish larvae were

randomly obtained for NMDA staining.

In vivo targeting effect of bacteria-mediated nanoparticle system. The *in vivo* targeting effects of Bac@Ceria were performed on Balb/c mice (n = 3). DiR labeled Bac (10^8 CFU mL^{-1}) and Bac-N_3 (10^8 CFU mL^{-1}) were mixed with Cy5.5 modified Ceria (50 μg mL^{-1}) and Ceria-DBCO (50 μg mL^{-1}) overnight, respectively. The mixture was washed with normal saline solution for three times (3920 g, 3 min). All mice were divided into 4 groups randomly. 100 μL Cy5.5 modified Ceria, 100 μL DiR labeled Bac was only orally administered. Bac-N_3 + Ceria-DBCO was injected intravenously, and in another group, Bac-N_3 + Ceria-DBCO was *p.o.* gavaged into mice. The fluorescence images were obtained *via* IVIS (Perkin Elmer). After 24 h injection, mice were sacrificed, main organs were collected to detect the bio-distribution of Bac@Ceria by IVIS system.

In vivo lead detoxification effect in chronic lead poisoning. The newborn Kunming mice were administered to 4 groups (n = 12), including saline, EDTA (*i.v.*, 15 mg kg^{-1}), DMSA (*oral*, 15 mg kg^{-1}), Bac@Ceria (*oral*, 50 μg kg^{-1}, 10^{10} CFU). During the first week, lead (1 mg kg^{-1}) was orally administered to mouse mothers per day. 7 days later, lead (1 mg kg^{-1}) was gavaged to each mouse per day. Several mice of each group were chosen for Morris water maze test at random. At the end of treatment, blood and main organs were collected for measuring lead concentration by ICP-MS, and the main organs were also obtained for for hematoxylin-eosin staining (H&E). The intestine tissues were collected to measure the content of myeloperoxidase (MPO).

In vivo lead detoxification effect in acute lead poisoning. The 5 week-old female Kunming mice were randomly divided into 5 groups (n = 6). Lead (100 mg kg^{-1}) was orally administered to each mouse per day. Mice of three groups were orally administered with saline, Bac (*oral*, 10^{10} CFU) and Bac@Ceria (*oral*, 50 μg kg^{-1}, 10^{10} CFU). Mice of another two groups were injected with EDTA (*i.v.*, 30 mg kg^{-1}) and Bac@Ceria (*i.v.*, 50 μg kg^{-1}, 10^7 CFU). After treatment, mice were sacrificed, blood and mian organs were collected for

measuring lead concentration by ICP-MS, and the intestine were also obtained for H&E staining. The MPO, SOD and GSH-px values in intestine tissues were analyzed.

In vivo biocompatibility in murine models. Female Balb/c mice (5 weeks, n = 3) were randomly divided into 4 groups for mice body temperature measure. saline, Bac@Ceria (*oral*, 50 μg kg^{-1}, 10^{11} CFU mL^{-1}, 100 μL), Bac@Ceria (*i.v.*, 50 μg kg^{-1}, 10^{11} CFU mL^{-1}, 100 μL), lipopolysaccharide (LPS, *i.v.*, 500 μg mL^{-1}, 100 μL) were administered into mice. The mice body temperature was measured with an electric thermometer (FR1DZ1, Microlife, Switzerland) at different time points. Next, female Balb/c mice (5 weeks, n = 3) were randomly divided into 4 groups for blood biochemical indexes. saline, EDTA (*i.v.*, 15 mg kg^{-1}), DMSA (*oral*, 15 mg kg^{-1}), Bac@Ceria (*oral*, 50 μg kg^{-1}, 10^{10} CFU) were administered into mice, respectively. The blood samples were obtained from heart (100 μL, per mouse) for blood biochemical indexes by biochemical auto analyzer (MNCHIP, Tianjin, China).

In vivo C-reactive protein (CRP) and procalcitonin (PCT) assay. Female Balb/c mice (5 weeks, n = 3) were randomly divided into 4 groups. Saline, Bac (*oral*, 10^{11} CFU mL^{-1}, 100 μL), Bac@Ceria (*i.v.*, 50 μg kg^{-1}, 10^{11} CFU mL^{-1}, 100 μL) and Bac@Ceria (*oral*, 50 μg kg^{-1}, 10^{11} CFU mL^{-1}, 100 μL) were administered into mice. 12 h later, mice were sacrificed, the blood were collected to measure the serum CRP and PCT concentrations.

Statistical analysis. Statistical analysis was performed using a Student's t test. The differences were considered to be statistically significant for a p value <0.05.

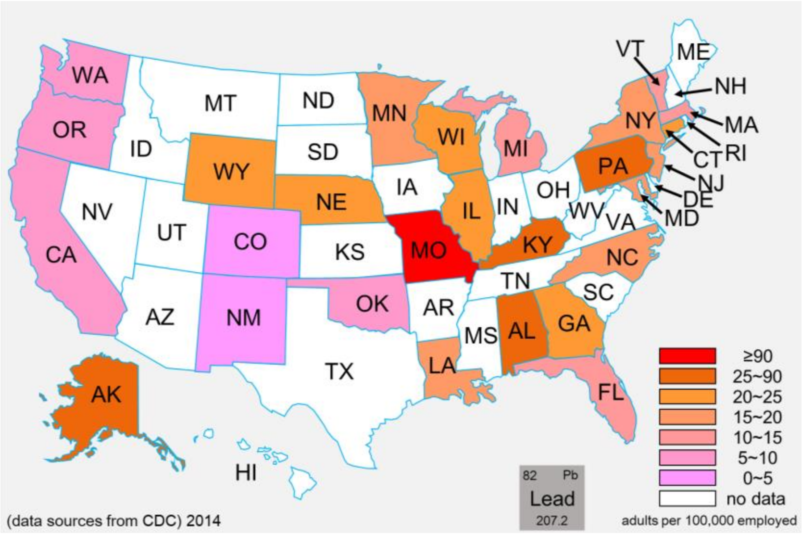

Figure S1 Distribution of the number of lead-poisoning patients in the USA (data sources from CDC, 2014).

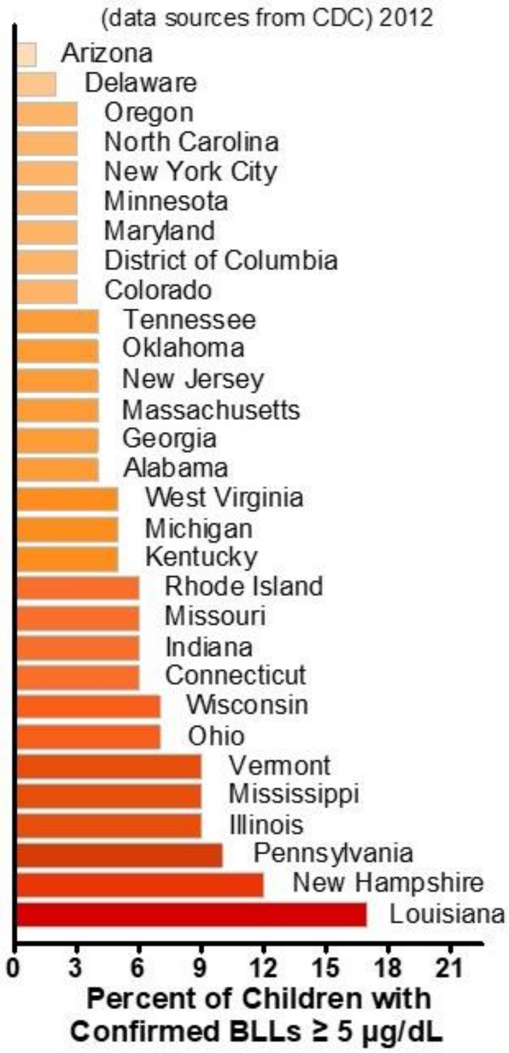

Figure S2 Levels of the blood lead in children at the USA (data sources from CDC, 2012).

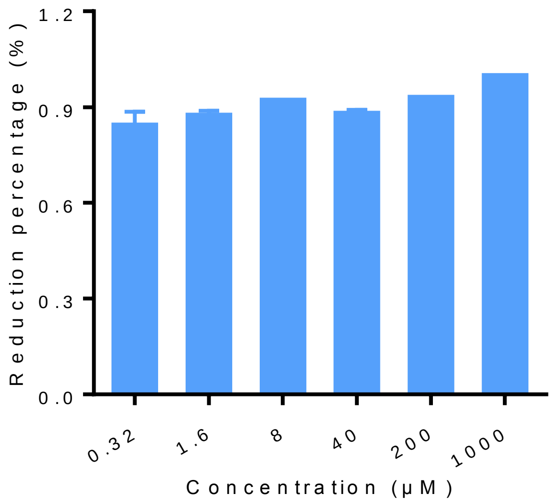

Figure S3 Effect of biodegradation rate of different concentration levels of lead.

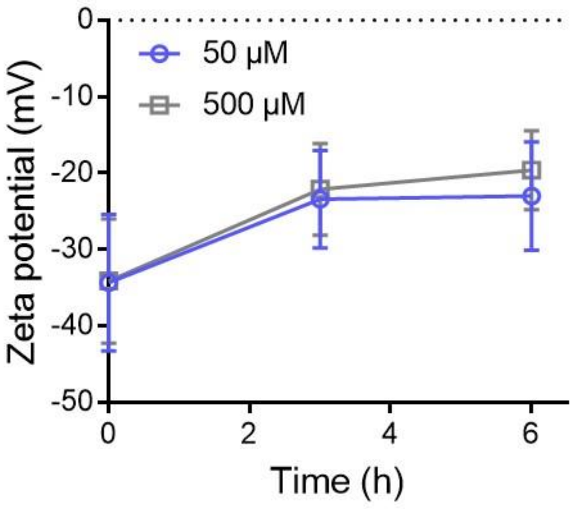

Figure S4 Zeta potential of bacteria after 50 μM and 500 μM lead exposure at different time points, respectively.

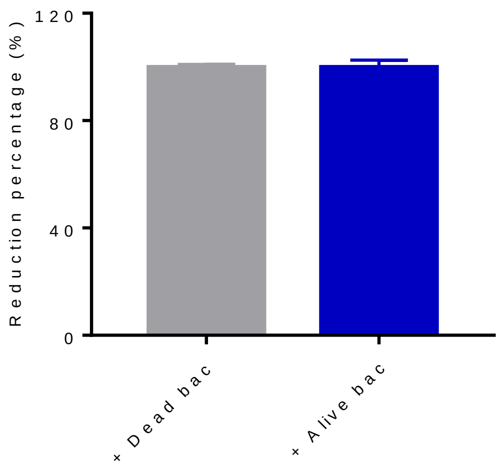

Figure S5 The reduction rate of lead after treatment of dead/alive bacteria.

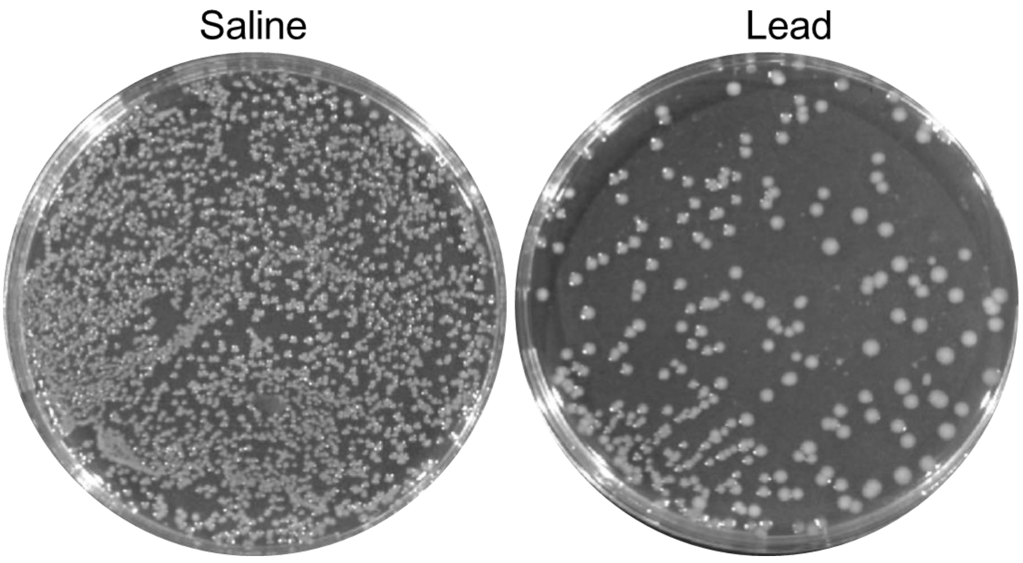

Figure S6 Relative viability of lead.

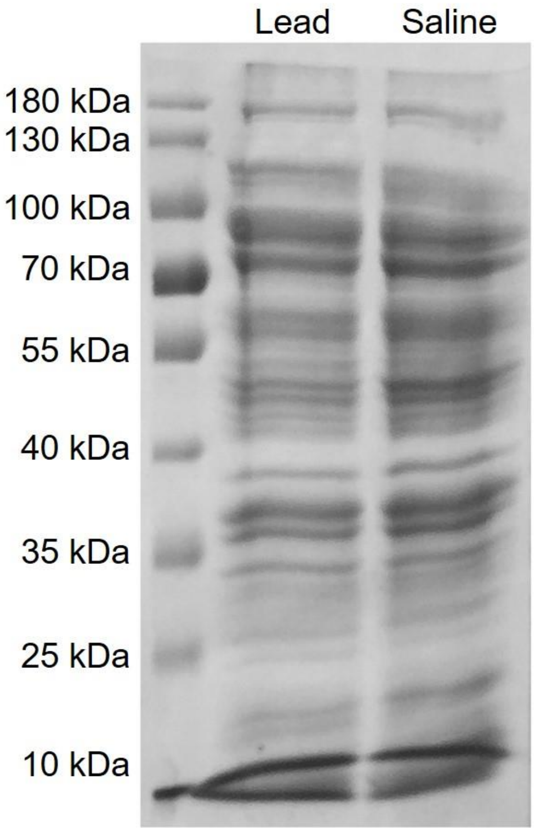

Figure S7 The protein analysis in bacteria after lead exposure by sulfate-polyacrylamide gel electrophoresis (SDS-PAGE).

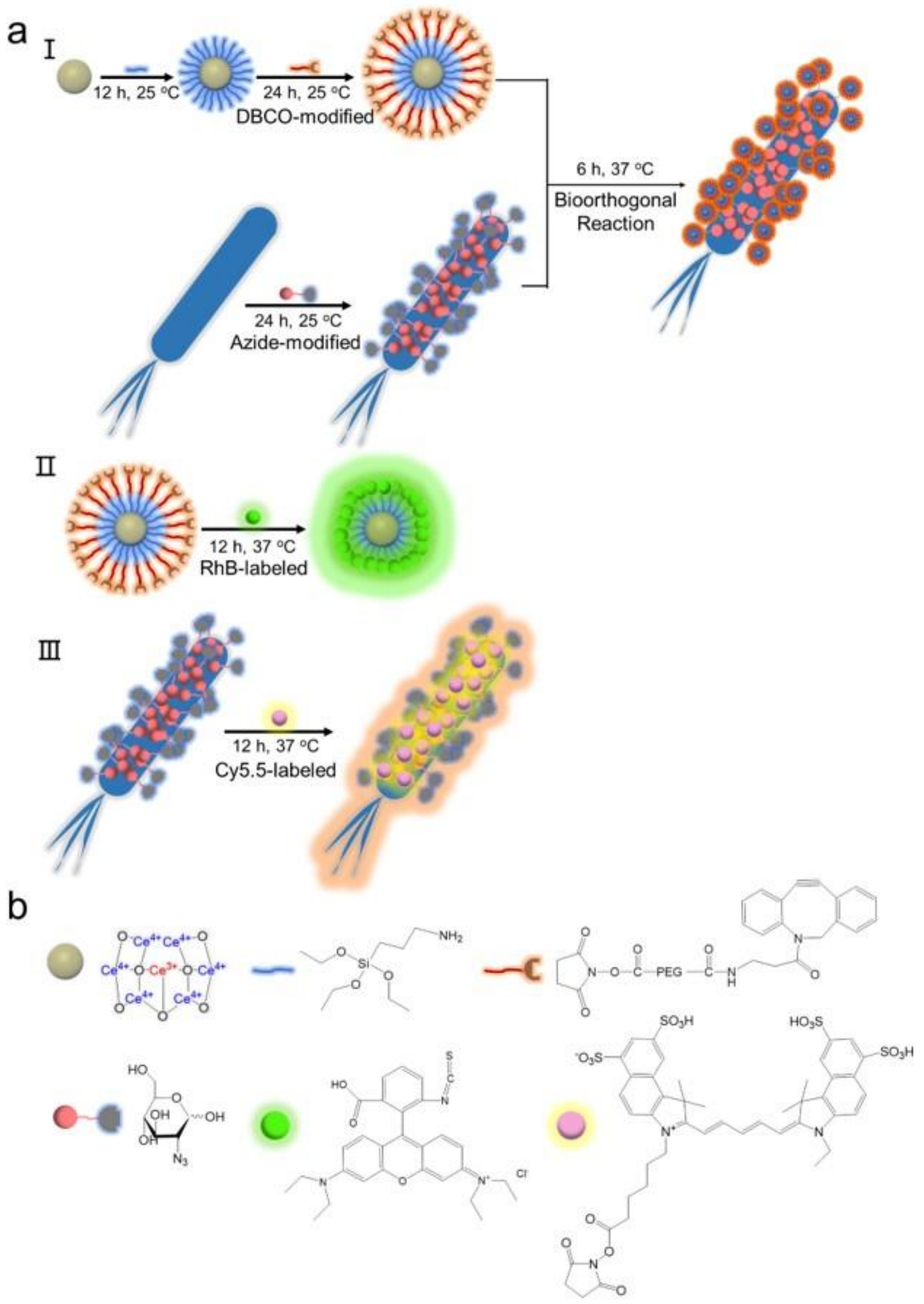

Figure S8 (a) Diagrammatic drawings for the synthesis of Bac@Ceria by bio-orthogonal reaction, the RhB-labeled Bac and the Cy5.5-labeled Ceria. (b) The structural formula of each component in (a).

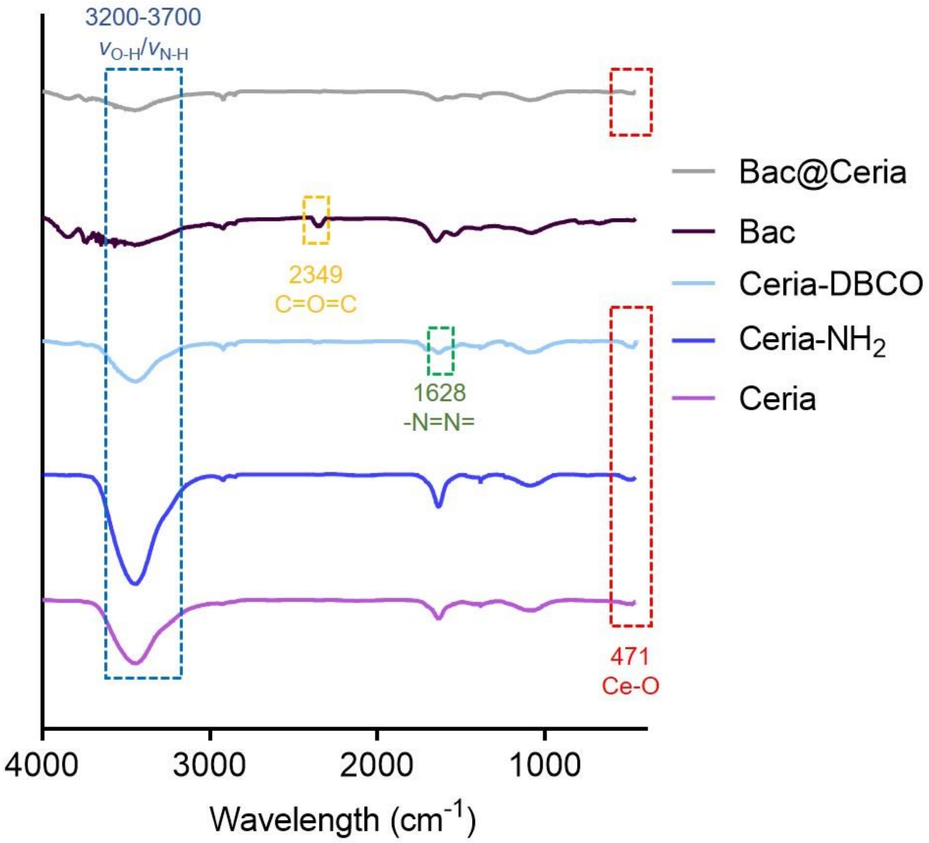

Figure S9 FT-IR spectrum of Ceria, Ceria-NH_2, Ceria-NH_2-DBCO, Bac and Bac@Ceria.

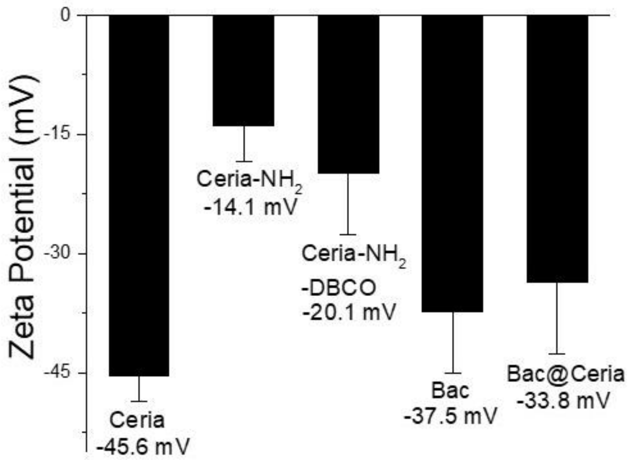

Figure S10 Zeta potential of Ceria, Ceria-NH_2, Ceria-NH_2-DBCO, Bac and Bac@Ceria.

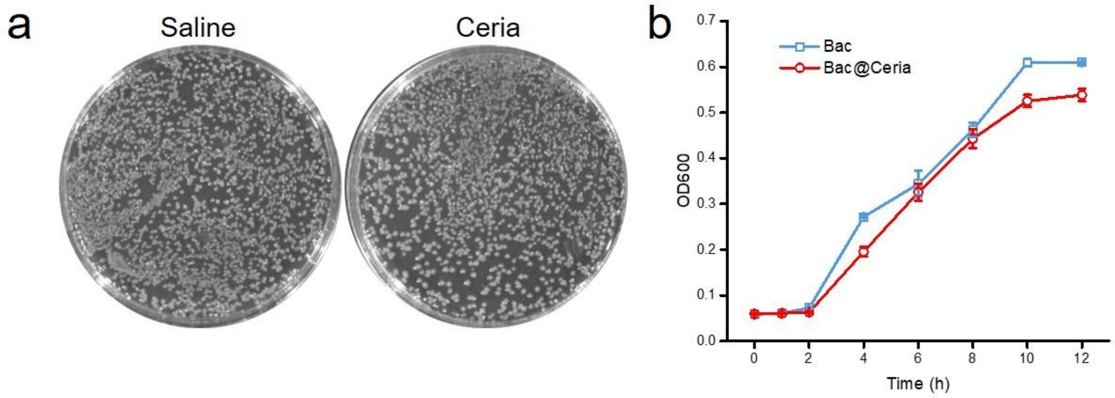

Figure S11 Relative viability of Bac after Ceria modified by (a) LB agar plates and (b) OD600 changes.

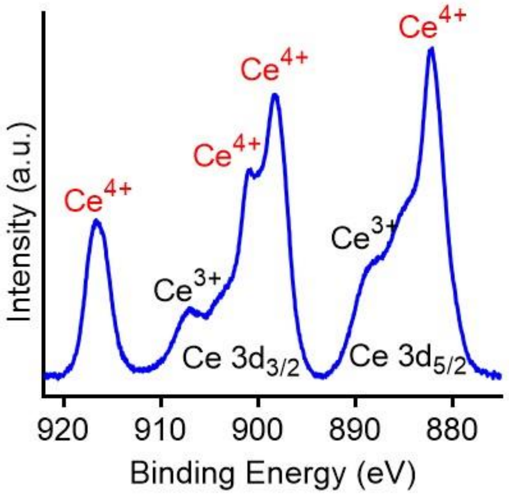

Figure S12 XPS spectrum of Ceria.

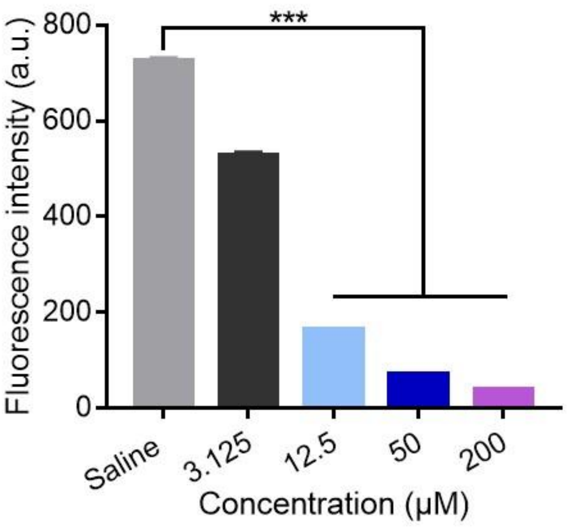

Figure S13 H_2O_2 degradation capability of various concentration Bac@Ceria by TA.

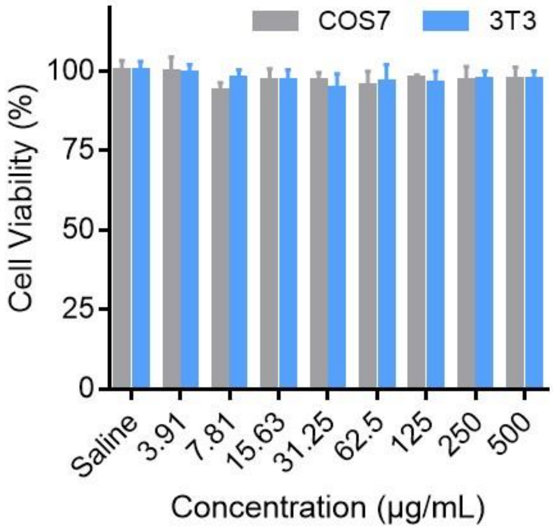

Figure S14 Cell viability of Ceria after co-inculcating 24 h.

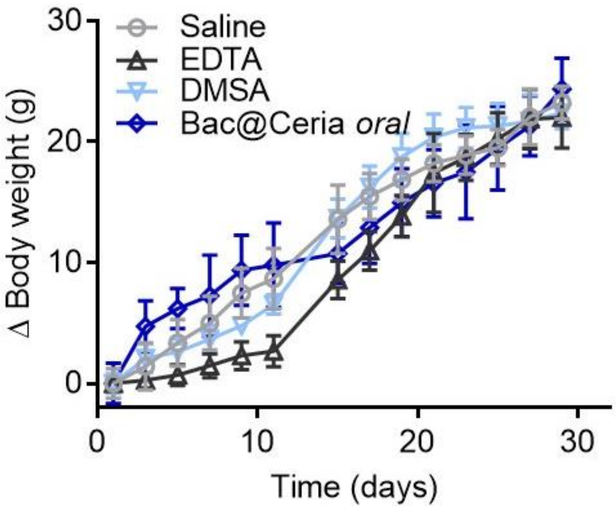

Figure S15 Change of chronic poisoning-mice weight after different treatments (n = 12).

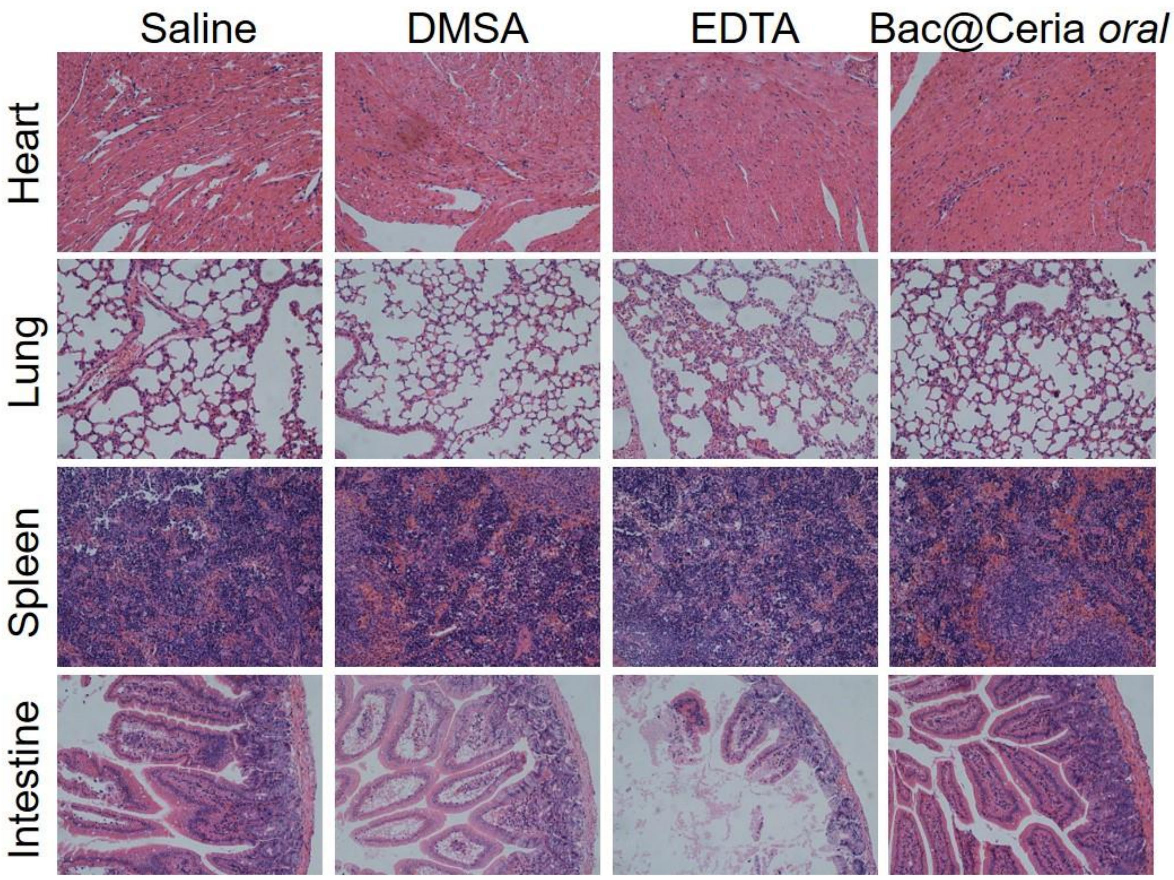

Figure S16 H&E staining of main organs in chronic poisoning-mice after different treatment. (40 ×)

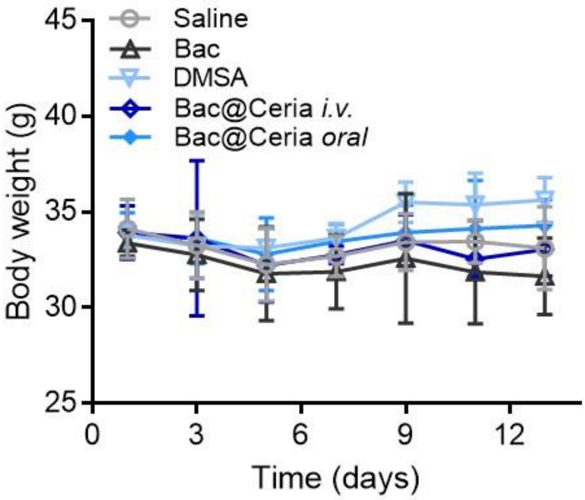

Figure S17 Mice weight of acute poisoning-mice after different treatment (n = 6).

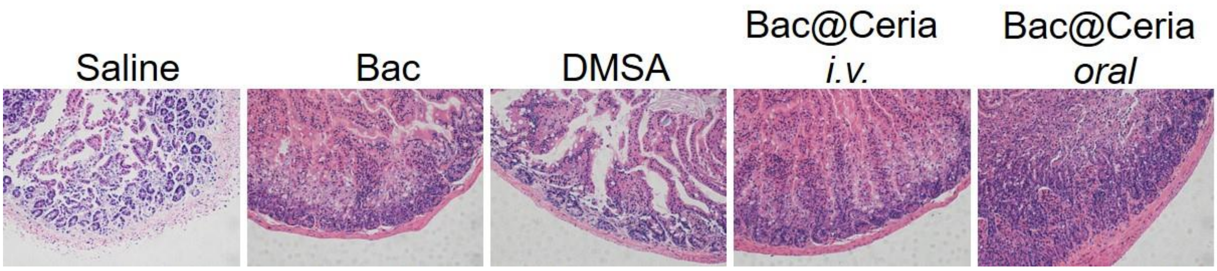

Figure S18 H&E staining of intestine in acute poisoning-mice after different treatment. (40 ×)

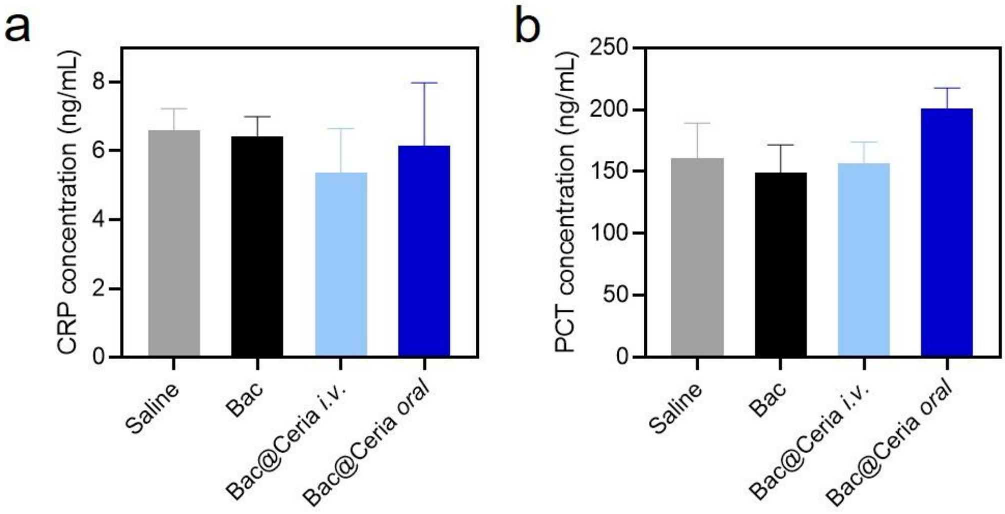

Figure S19 The concentrations of (a) C-reaction protein (CRP) and (b) procalcitonin (PCT). (n = 3).
